# Physiological and Proteomic Responses of the Tetraploid *Robinia pseudoacacia* L. to High CO_2_ Levels

**DOI:** 10.3390/ijms25105262

**Published:** 2024-05-11

**Authors:** Jianxin Li, Subin Zhang, Pei Lei, Liyong Guo, Xiyang Zhao, Fanjuan Meng

**Affiliations:** 1 College of Forestry and Grassland, Jilin Agriculture University, Changchun 130118, China; ljxin1022@163.com (J.L.); 18800465845@163.com (P.L.); 2 College of Life Science, Northeast Forestry University, Harbin 150040, China; zhbin972@163.com (S.Z.); 18512231143@163.com (L.G.); 3 Jilin Provincial Key Laboratory of Tree and Grass Genetics and Breeding, Changchun 130118, China

**Keywords:** *Robinia pseudoacacia* L., high CO_2_, photosynthesis, respiration, proteomic, stomatal

## Abstract

The increase in atmospheric CO_2_ concentration is a significant factor in triggering global warming. CO_2_ is essential for plant photosynthesis, but excessive CO_2_ can negatively impact photosynthesis and its associated physiological and biochemical processes. The tetraploid *Robinia pseudoacacia* L., a superior and improved variety, exhibits high tolerance to abiotic stress. In this study, we investigated the physiological and proteomic response mechanisms of the tetraploid *R. pseudoacacia* under high CO_2_ treatment. The results of our physiological and biochemical analyses revealed that a 5% high concentration of CO_2_ hindered the growth and development of the tetraploid *R. pseudoacacia* and caused severe damage to the leaves. Additionally, it significantly reduced photosynthetic parameters such as *P*n, *G*s, *T*r, and *C*i, as well as respiration. The levels of chlorophyll (Chl a and b) and the fluorescent parameters of chlorophyll (*Fm*, *Fv*/*Fm*, *q*P, and *ETR*) also significantly decreased. Conversely, the levels of ROS (H_2_O_2_ and O_2_^·−^) were significantly increased, while the activities of antioxidant enzymes (SOD, CAT, GR, and APX) were significantly decreased. Furthermore, high CO_2_ induced stomatal closure by promoting the accumulation of ROS and NO in guard cells. Through a proteomic analysis, we identified a total of 1652 DAPs after high CO_2_ treatment. GO functional annotation revealed that these DAPs were mainly associated with redox activity, catalytic activity, and ion binding. KEGG analysis showed an enrichment of DAPs in metabolic pathways, secondary metabolite biosynthesis, amino acid biosynthesis, and photosynthetic pathways. Overall, our study provides valuable insights into the adaptation mechanisms of the tetraploid *R. pseudoacacia* to high CO_2_.

## 1. Introduction

CO_2_ is a crucial substrate for plant photosynthesis. The levels of atmospheric CO_2_ have a significant impact on plant growth, development, and biomass [1]. Since the industrial era, atmospheric CO_2_ concentrations have increased by more than 40%, and the current ambient CO_2_ concentrations exceed 417 ppm [2]. The rise in CO_2_ concentration has resulted in significant alterations in global temperatures, intensifying the greenhouse effect and exposing plants to elevated CO_2_ levels, higher temperatures, and drought. Consequently, this poses a considerable challenge to plant growth and reproduction. The impact of CO_2_ enrichment on growth and yield for C3 plants can vary and may be influenced by species-specific factors [3].

Previous studies have consistently shown that higher levels of CO_2_ have a significant impact on the physiological functioning of plants [4,5,6]. On the one hand, elevated CO_2_ directly affects plant photosynthesis. Increased atmospheric CO_2_ concentrations lead to a higher number of leaves, longer branches, and greater biomass while also causing a decrease in stomatal density. Additionally, it can result in visible symptoms such as wilting and drooping leaves [3,7]. According to previous studies, the net photosynthetic rate and water use efficiency (WUE) of *A. marina* and *R. stylosa* increased when exposed to a slight elevation in CO_2_ concentration. These findings suggest that the promotion of photosynthesis was facilitated by the elevated CO_2_ concentration [7]. Furthermore, it has been observed that rising CO_2_ concentrations contribute to an increase in phytoplankton growth rate and lipid productivity [8]. Another study found that corn exhibits higher biomass and leaf area, as well as enhanced starch synthesis, when exposed to elevated CO_2_ concentrations [9]. The Rubisco content and light-saturated photosynthetic rate in plant leaves were observed to be significantly reduced in a high CO_2_ environment [10]. Similarly, when *Pinus sylvestris* was exposed to this environment for an extended period, both the stomatal conductance and total stomatal number showed a significant decrease [11]. On the other hand, in *Allium sativum* L., the elevated CO_2_ atmosphere had a contrasting effect, significantly enhancing the activity of pyruvate decarboxylase (PDC) and alcohol dehydrogenase (ADH). This, in turn, caused a substantial increase in the content of alcohols, aldehydes, and phenols, resulting in toxic effects [12]. On the contrary, research has demonstrated that high CO_2_ levels can also actually mitigate ozone-induced oxidative damage in wheat [8]. Additionally, the impact of CO_2_ concentrations varies depending on the plant species. For example, in the case of reticulated melons, low CO_2_ has been found to increase both the initial and maximum photosynthetic efficiency. However, when exposed to high CO_2_, the maximum photosynthetic efficiency of reticulated melons actually declines [13].

On the other hand, previous studies have demonstrated that CO_2_-induced stomatal closure is dependent on Ca^2+^ and that elevated CO_2_ levels stimulate an increase in free Ca^2+^ concentration in defense cells [14,15,16,17]. Stomata, which are small pores on the leaf surface, play a crucial role in regulating the uptake of carbon dioxide for photosynthesis and the loss of water through transpiration [18]. The movement of stomata is influenced by various environmental factors, including CO_2_ concentration. Stomatal opening is promoted by low concentrations of CO_2_, while stomatal closure is induced by high concentrations of CO_2_. In the guard cells of *Arabidopsis thaliana*, the carbonic anhydrases *β*CA1 and *β*CA4 are involved in mediating CO_2_-controlled stomatal movement and development [19]. Additionally, BIG proteins play a critical role in inhibiting stomatal opening and promoting stomatal closure in response to CO_2_ [20]. Reactive oxygen species (ROS) and nitric oxide (NO) are important signaling molecules that regulate stomatal movement in plants [21]. It has been shown that CO_2_-mediated stomatal closure requires the generation of ROS, which has an important role in the regulation of stomatal aperture [22,23]. NO plays a role in both abscisic acid (ABA)- and Ca^2+^-mediated stomatal closure and is located downstream of ROS, an important participant in the stomatal closure process [24,25]. In *A. thaliana*, ROS are mainly produced by NADPH oxidase and peroxidase at the plasma membrane [26,27], while NO is mainly produced by nitrate reductase (NR) and NOA1 and induces stomatal closure [28].

Plant respiration is a significant metabolic process on a global scale, accounting for approximately 40–60% of the rate of carbon assimilation [29]. Mitochondria, which are essential organelles involved in cellular material and energy metabolism, possess two respiratory pathways on their inner mitochondrial membrane [30]. There are two pathways involved in respiration: the cytochrome pathway, which is found in nearly all organisms, and the alternate respiration pathway. The alternate respiration pathway is activated when the cytochrome pathway experiences stress conditions like low temperature, drought, ozone, and high salinity soils [31]. The impact of CO_2_ on mitochondrial respiration is of great significance in the investigation of plant respiratory metabolism. Previous studies have demonstrated that there exists an alternate respiration pathway, which is insensitive to cyanide (CN) but sensitive to SHAM and is facilitated by alternate oxidase (AOX) [32,33,34]. Additionally, pyruvate, a crucial metabolite in organisms, has been found to have a notable enhancing effect on the activity of AOX [35,36,37].

The respiration rate serves as a primary indicator of plant respiratory metabolism. By examining the respiration rate of plants, we can directly observe the impact of high CO_2_ concentration on plant respiratory metabolism. However, the effect of elevated CO_2_ concentration on the plant respiration rate varies depending on the specific plants and environmental conditions. It has been discovered that elevated CO_2_ concentrations resulted in an 8.4% decrease in the daily respiration rate of wheat and sunflower, as well as a 16.2% decrease in the respiration rate during darkness. This could be attributed to the reduction in leaf nitrogen content and the downregulation of photosynthesis caused by elevated CO_2_ concentrations, which subsequently leads to a decrease in plant respiration rate [38]. However, previous studies have demonstrated that elevated CO_2_ concentrations have the potential to enhance respiratory substrate utilization, upregulate respiratory genes, and increase the number of mitochondria. As a result, this leads to an overall increase in the rate of plant respiration [39,40].

The tetraploid *R. pseudoacacia* L. is a deciduous ornamental tree species belonging to the genus *R. pseudoacacia* in the family Leguminosae. It has four sets of chromosomes (4n = 44) and was created through the application of colchicine to the diploid *R. pseudoacacia* (2n = 22) of a homologous species. This tetraploid species was introduced to China from South Korea [41]. Compared to the diploid *R. pseudoacacia*, the tetraploid *R. pseudoacacia* is considered an excellent variety due to its higher yield, faster growth rate, larger leaves, and numerous outstanding characteristics such as drought resistance, saline resistance, low-temperature resistance, and heat resistance [42,43,44,45]. The tetraploid *R. pseudoacacia* exhibits a robust root system, strong adaptability to various environments, and a high capacity for photosynthetic carbon sequestration [46]. In light of prevalent environmental pollution and global climate change, it is imperative to investigate the effects of high CO_2_ levels on the physiological processes of the tetraploid *R. pseudoacacia*.

Plants display distinct physiological and biochemical traits when subjected to prolonged exposure to elevated CO_2_ concentrations. This study focuses on the tetraploid *R. pseudoacacia* as the experimental material to examine the stomatal movement pattern and physiological alterations in photosynthesis and respiration in response to high CO_2_ conditions. Additionally, a proteomic approach was employed to identify crucial regulatory networks and proteins associated with the response of the tetraploid *R. pseudoacacia* to high CO_2_ treatment. The objective of this research is to gain insights into the mechanisms by which plants respond to high CO_2_ environments.

## 2. Results

### 2.1. Response of Morphological and Gas Exchange Parameters of Tetraploid R. pseudoacacia to High CO_2_

**Figure 1:**
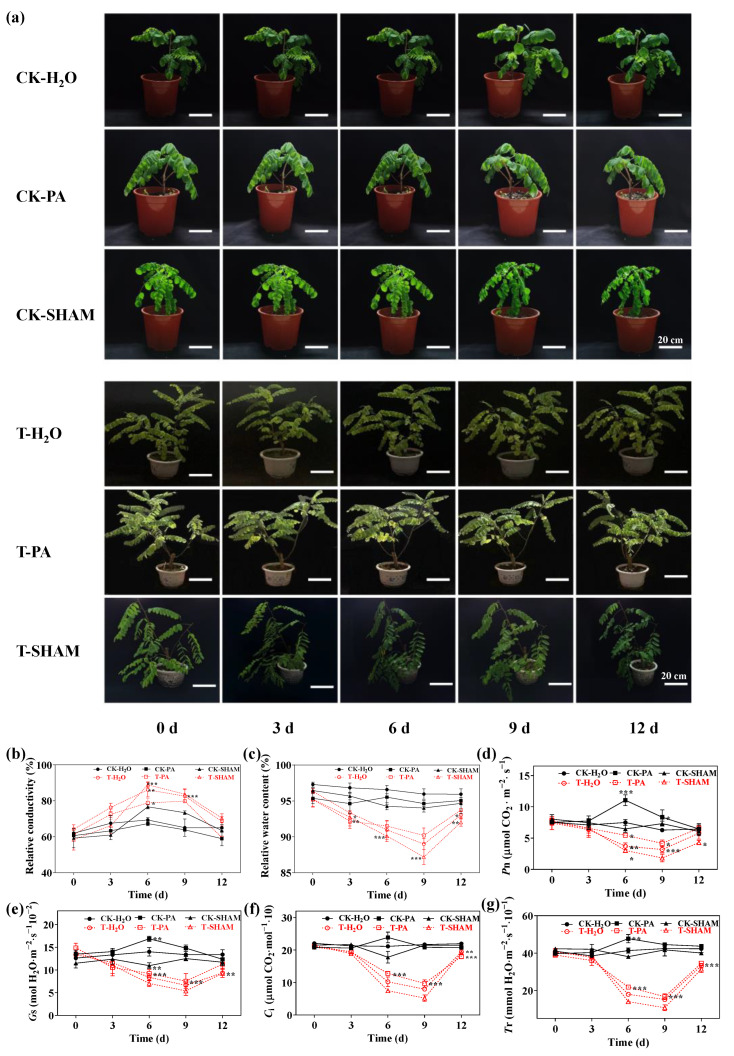
Physiological response of the tetraploid *R. pseudoacacia* to high CO_2_ concentrations. (**a**) Phenotypes of *R. pseudoacacia* under CO_2_ treatment. Plants were exposed to CO_2_ stress for 9 days and then recovered to air CO_2_ levels for 3 days. CK-H_2_O: control; CK-PA: pyruvic acid-treated; CK-SHAM: salicylhydroxamic acid-treated; T-H_2_O: CO_2_-treated; T-PA: co-treated with CO_2_ and PA; and T-SHAM: co-treated with CO_2_ and SHAM. Bar: 20 cm. Effect of high CO_2_ concentrations on relative electrical conductivity (**b**), relative water content (**c**), net photosynthetic rate (*P*n) (**d**), stomatal conductance (*G*s) (**e**), intercellular CO_2_ concentration (*C*i) (**f**), and transpiration rate (*T*r) (**g**). Six biological replicates were analyzed, and the error bars represent the SE. Asterisks indicate significant differences as determined by Dunnett’s test. (* *p* ≤ 0.05; ** *p* ≤ 0.01; *** *p* ≤ 0.001).

Under normal CO_2_ conditions, the growth of the tetraploid *R. pseudoacacia* was uniform and healthy (CK-H_2_O). When the respiratory inhibitor SHAM was added (CK-SHAM), the REC increased, and the RWC of the leaves decreased. However, supplementation with the respiratory enhancer PA (CK-PA) did not cause significant changes in REC and RWC compared to the control. After 9 days of treatment with a 5% CO_2_ concentration, the leaves exhibited wilting, drooping, yellowing, and even abscission at the apex (T-H_2_O). The yellowing and abscission of leaves worsened with the addition of SHAM (T-SHAM), while the wilting was alleviated with the addition of PA (T-PA) (Figure 1a). After 6 days of treatment, the REC of the CO_2_-treated leaves significantly increased by 34.2%, 27.2%, and 52.2% in T-H_2_O, T-PA, and T-SHAM, respectively (Figure 1b). However, the RWC of T-H_2_O, T-PA, and T-SHAM significantly decreased by 7.6%, 5.3%, and 8.4%, respectively, after 9 days of CO_2_ treatment (Figure 1c).

Under normal CO_2_ conditions, the presence of PA did not have a significant effect on the photosynthetic parameters (*P*n, *G*s, *C*i, and *T*r) of the tetraploid *R. pseudoacacia*’s leaves compared to the control (CK-H_2_O). However, after supplementation with SHAM on the 6th day, there was a slight fluctuation and decrease in *P*n, *G*s, and *T*r (excluding *C*i) compared to the control. When exposed to high CO_2_, the photosynthetic parameters of the leaves (*P*n, *G*s, *C*i, and *T*r) significantly decreased compared to the control (CK-H_2_O), reaching the lowest values on the 9th day. *P*n, *G*s, *C*i, and *T*r were 49.5%, 50.5%, 63%, and 63.7% lower than the control, respectively. After the high CO_2_ treatment, *R. pseudoacacia* showed some recovery for three days under normal CO_2_ conditions, with an increase in the photosynthetic parameters. However, these parameters were consistently lower than the control (Figure 1d–g). In conclusion, the results indicate that the photosynthetic gas parameters of *R. pseudoacacia* leaves significantly decreased after exposure to high CO_2_. The addition of the respiration enhancer PA mitigated the inhibition of photosynthesis caused by high CO_2_, while the addition of the respiration inhibitor SHAM enhanced the inhibition of photosynthesis by high CO_2_.

### 2.2. Response of Antioxidant System of Tetraploid R. pseudoacacia to High CO_2_

**Figure 2:**
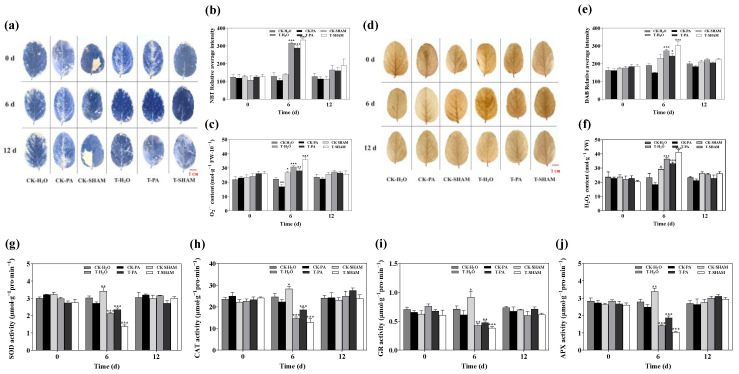
Effect of high CO_2_ on NBT staining of the fresh leaves (**a**,**b**), O_2_^·−^ level (**c**), DAB staining of the fresh leaves (**d**,**e**), H_2_O_2_ content (**f**), SOD (**g**), CAT (**h**), GR (**i**), and APX (**j**). Bar: 1 cm. Six biological replicates were analyzed, and the error bars represent the SE. Asterisks indicate significant differences as determined by Dunnett’s test. (* *p* ≤ 0.05; ** *p* ≤ 0.01; *** *p* ≤ 0.001).

To investigate the impact of high CO_2_ on the antioxidant system of the tetraploid *R. pseudoacacia*, we initially measured the levels of H_2_O_2_ and O_2_^·−^. Under normal CO_2_ conditions, the levels of H_2_O_2_ and O_2_^·−^ decreased after PA treatment compared to CK-H_2_O, whereas the levels of H_2_O_2_ and O_2_^·−^ increased after SHAM supplementation. After 6 days of high CO_2_ treatment, the levels of H_2_O_2_ and O_2_^·−^ in the leaves were significantly higher than in CK-H_2_O. Furthermore, after SHAM supplementation, the levels of H_2_O_2_ and O_2_^·−^ were further increased compared to T-H_2_O. On the other hand, after PA treatment, the levels of H_2_O_2_ and O_2_^·−^ were slightly decreased compared to T-H_2_O but were still higher than CK-H_2_O (Figure 2c,f). In addition, there was no significant difference in the staining intensity of DAB and NBT on the leaves before CO_2_ treatment. However, after 6 days of high CO_2_ treatment, the mean staining intensities of DAB and NBT on the leaves were significantly higher compared to CK-H_2_O. Similarly, the SHAM treatment increased the mean staining intensity, while PA treatment decreased it compared to T-H_2_O (Figure 2a–f).

SOD and CAT are considered the most significant enzymes in the plant antioxidant system, as their activity levels indicate the extent of damage caused by external factors. Meanwhile, GR and APX play a crucial role in maintaining the balance of the ASA-DHA cycle. APX functions to eliminate the excessive accumulation of H_2_O_2_ in a plant, while GR is primarily responsible for converting oxidized glutathione to reduced glutathione. When CO_2_ conditions are normal, the addition of SHAM slightly enhances the activities of SOD, CAT, GR, and APX. On the other hand, the supplementation of PA does not lead to any significant changes in the activities of these enzymes. However, under high CO_2_ levels, the activities of SOD, CAT, GR, and APX decrease. Specifically, in T-H_2_O, T-PA, and T-SHAM, SOD activity decreased by 29.7%, 22.4%, and 54.3%, respectively, CAT activity decreased by 40.3%, 24.1%, and 47.8%, respectively, GR activity decreased by 39.6%, 32.6%, and 46%, respectively, and APX activity decreased by 49.2%, 32.8%, and 62.7%, respectively (Figure 2g–j).

### 2.3. Changes in Chlorophyll Levels and Fluorescence Parameters

**Figure 3:**
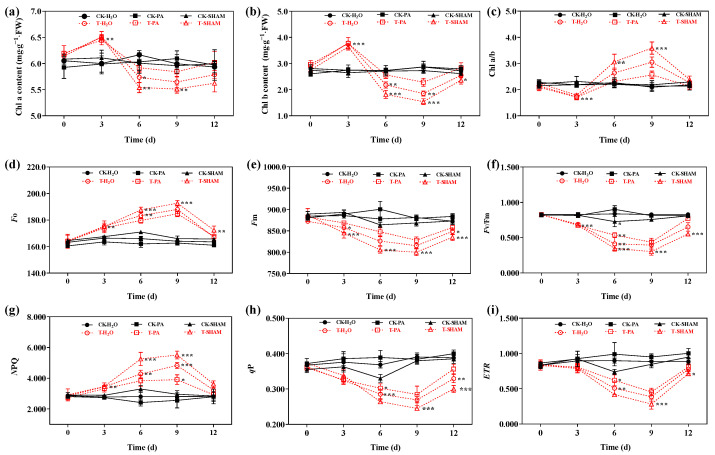
Effect of high CO_2_ concentration on chlorophyll content and chlorophyll fluorescence parameters in *R. pseudoacacia*. Chl a content (**a**), Chl b content (**b**), Chl a/b (**c**), minimal fluorescence (*Fo*) (**d**), maximal fluorescence (*Fm*) (**e**), primary light energy conversion efficiency of PSII (*Fv/Fm*) (**f**), nonphotochemical quenching (NPQ) (**g**), photochemical quenching (*q*P) (**h**), and relative electron transport rate *(ETR*) (**i**). Six biological replicates were analyzed, and the error bars represent the SE. Asterisks indicate significant differences as determined by Dunnett’s test. (* *p* ≤ 0.05; ** *p* ≤ 0.01; *** *p* ≤ 0.001).

Under normal CO_2_ conditions, there were no significant differences in the content of Chl a and Chl b, as well as the ratio of Chl a/b, among the CK-H_2_O, CK-PA, and CK-SHAM groups. However, in the high CO_2_ treatment group, the levels of Chl a and Chl b increased, but the ratio of Chl a/b decreased compared to CK-H_2_O after 3 days. In the CK-PA and CK-SHAM groups, there was a significant decrease in the levels of Chl a and Chl b, while the ratio of Chl a/b significantly increased after 6–9 days. Interestingly, the T-SHAM group had lower levels of Chl a and Chl b, whereas the T-PA group had higher levels of Chl a and Chl b (Figure 3a–c).

Under normal CO_2_ conditions, the leaf chlorophyll fluorescence parameters did not show a significant response to supplementation with SHAM and PA compared to the control. However, after 9 days of high CO_2_ treatment, *Fo* and NPQ increased by 15.6% and 63.4%, respectively. *Fo* and NPQ were higher than in T-H_2_O after supplementation with SHAM but lower than in T-H_2_O after supplementation with PA. Upon restoration of normal CO_2_ conditions, *Fo* and NPQ returned to the control values (Figure 3d,g). During the high CO_2_ treatment, *Fm*, *Fv/Fm*, *q*P, and *ETR* exhibited a decreasing trend and were significantly different compared to CK-H_2_O. On day 9 of treatment, *Fm*, *Fv/Fm*, *q*P, and *ETR* decreased by 7.3%, 51.3%, 31% and 56.5%, respectively. *Fm*, *Fv/Fm*, *q*P, and *ETR* showed a more significant decrease with SHAM supplementation compared to T-H_2_O, while the addition of the accelerator increased *Fm*, *Fv/Fm*, *q*P, and *ETR* (Figure 3e,f,h,i). These results indicate that high CO_2_ has a severe impact on the chlorophyll levels and fluorescence parameters of the tetraploid *R. pseudoacacia*.

### 2.4. Effect of High CO_2_ on Leaf Respiration Parameters

**Figure 4:**
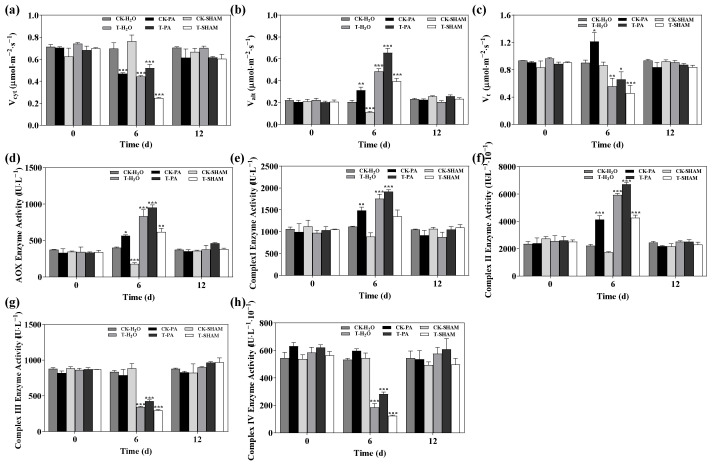
Effect of high CO_2_ concentration on respiratory parameters and mitochondrial electron transport chain complex enzyme activities in *R. pseudoacacia*. Cytochrome pathway capacity (V_cyt_) (**a**), alternative pathway capacity (V_alt_) (**b**), total respiration (V_t_) (**c**), AOX enzyme activity (**d**), complex I enzyme activity (**e**), complex II enzyme activity (**f**), complex III enzyme activity (**g**), and complex IV enzyme activity (**h**). Six biological replicates were analyzed, and the error bars represent the SE. Asterisks indicate significant differences as determined by Dunnett’s test. (* *p* ≤ 0.05; ** *p* ≤ 0.01; *** *p* ≤ 0.001).

Under normal CO_2_ conditions, the presence of PA contributed to an increase in V_alt_ and V_t_, while the addition of SHAM inhibited the increase in V_cyt_, V_alt_, and V_t_. After 6 days of high CO_2_ treatment, there was a rapid increase in Valt but a significant decrease in V_cyt_ and V_t_ compared to CK-H_2_O. When compared to T-H_2_O, V_cyt_, V_alt_, and V_t_ increased with PA supplementation but decreased with SHAM supplementation. Upon restoring normal CO_2_ conditions, there were no significant differences in V_cyt_, V_alt_, and V_t_ compared to the control (Figure 4a–c).

The enzyme activities of AOX, complex I, and complex II were increased by PA compared to CK-H_2_O. No significant changes in the enzyme activities of AOX, complex I, and complex II were observed after supplementation with SHAM under normal CO_2_ conditions. However, the enzyme activities of AOX, complex I, and complex II were significantly increased under high CO_2_ treatment. Furthermore, PA supplementation led to a more significant increase in the enzyme activities of AOX, complex I, and complex II compared to T-H_2_O, while the SHAM treatment also resulted in a significant increase in the enzyme activities of AOX, complex I, and complex II (Figure 4d–f). There were no significant changes in the enzyme activities of complexes III and IV, including after PA and SHAM supplementation, under normal CO_2_ conditions. However, under high CO_2_ conditions, the enzyme activities of complexes III and IV were significantly reduced. PA increased the enzyme activities of complexes III and IV compared to T-H_2_O, while SHAM decreased the enzyme activities of complexes III and IV. After restoration of normal CO_2_ conditions, the mitochondrial electron transport chain enzyme activities were not significantly different from those of the control (Figure 4g,h).

### 2.5. Effect of High CO_2_ on Leaf Stomatal Movement

**Figure 5:**
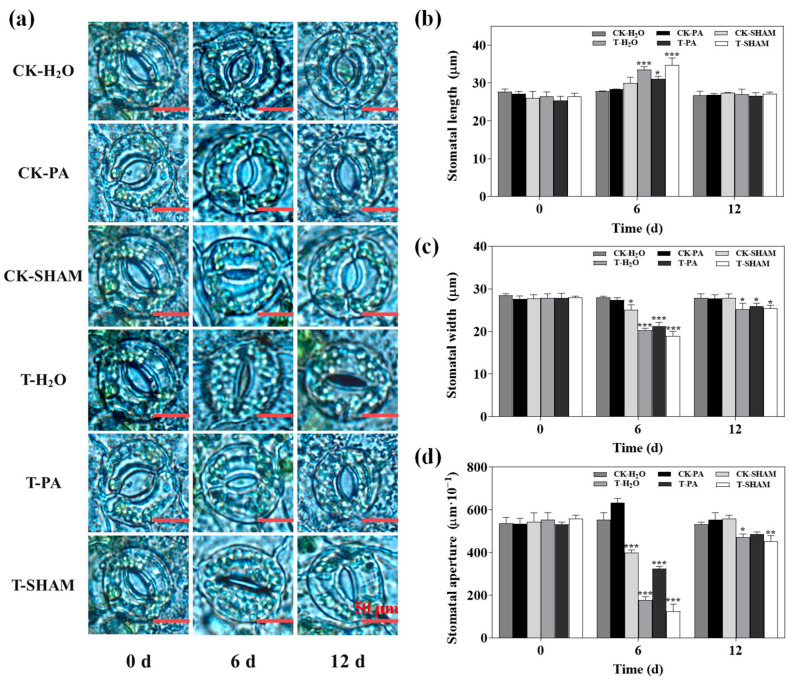
Effects of high CO_2_ concentration on stomatal morphology (**a**), stomatal length (**b**), stomatal width (**c**), and stomatal aperture of plant (**d**). Bar: 50 μm. Six biological replicates were analyzed, and the error bars represent the SE. Asterisks indicate significant differences as determined by Dunnett’s test. (* *p* ≤ 0.05; ** *p* ≤ 0.01; *** *p* ≤ 0.001).

To investigate the movement of stomata in the tetraploid *R. pseudoacacia* under high CO_2_ conditions, we conducted observations on stomatal morphology and counted the stomatal apertures. When exposed to normal CO_2_ levels, SHAM caused stomata closure, while PA had a lesser effect on the stomata and did not significantly change the stomatal aperture compared to CK-H_2_O. However, under the high CO_2_ treatment, T-H_2_O showed a 67.9% reduction in stomatal apertures compared to CK-H_2_O. Additionally, supplementation with SHAM further decreased the stomatal apertures by 30% compared to T-H_2_O, whereas supplementation with PA increased the stomatal apertures by 81.7% (Figure 5).

**Figure 6:**
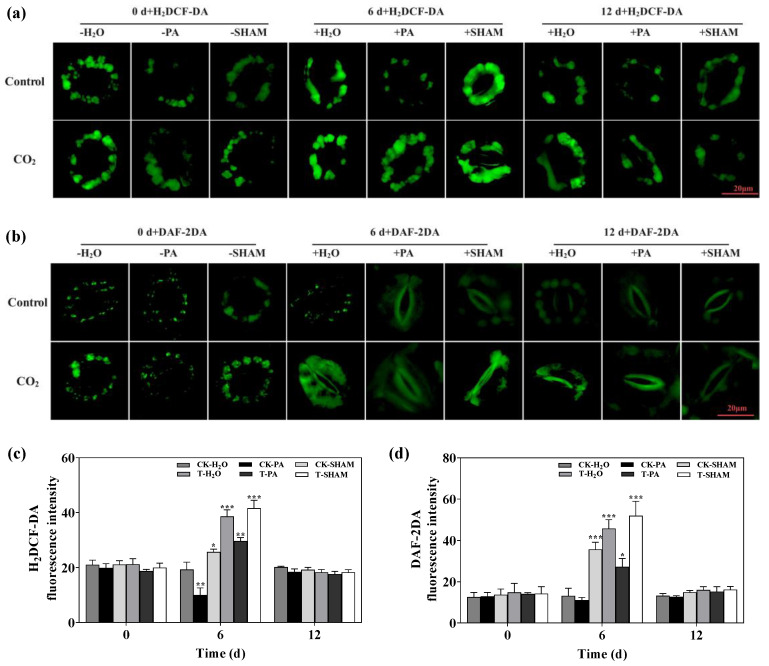
2′,7′-Dichlorodihydrofluorescein diacetate (H_2_DCF-DA) (**a**,**c**) and 5,6-Diaminofluorescein diacetate (DAF-2DA) (**b**,**d**) staining of guard cells under high CO_2_ conditions. Ten biological replicates were analyzed, and the error bars represent the SE. Asterisks indicate significant differences as determined by Dunnett’s test. (* *p* ≤ 0.05; ** *p* ≤ 0.01; *** *p* ≤ 0.001).

The fluorescence staining intensity of the guard cells was significantly enhanced after high CO_2_ treatment, as observed through the use of the fluorescent probes H_2_DCF-DA and DAF-2DA. This indicates that high CO_2_ treatment increased the accumulation of ROS and NO in the guard cells. Supplementation with SHAM further increased the fluorescence staining intensity, suggesting an increase in the accumulation of ROS and NO. On the other hand, supplementation with PA decreased the fluorescence staining intensity, indicating a decrease in the accumulation of ROS and NO in the guard cells (Figure 6). In conclusion, the tetraploid *R. pseudoacacia* regulates stomatal closure in response to a high CO_2_ environment by inducing the accumulation of ROS and NO in the guard cells.

### 2.6. Proteomic Analysis of Tetraploid R. pseudoacacia Based on High CO_2_ Conditions

To investigate the response of the tetraploid *R. pseudoacacia* to high CO_2_ levels, we conducted a proteomic analysis on six groups of *R. pseudoacacia* leaves (CK-H_2_O, CK-PA, CK-SHAM, T-H_2_O, T-PA, and T-SHAM). The quality of the samples was confirmed by analyzing the total ion chromatograms, which showed uniform peaks (Figure S1). Additionally, the PCA analysis indicated low variability among samples within each group, as the distribution of the three biological replicates was relatively concentrated. Two-dimensional plots demonstrated significant differences in the principal components between the high CO_2_ treatment groups and the groups under normal CO_2_ conditions (Figure S2). A total of 1652 proteins were quantified below the protein threshold of the 1.0% FDR criterion. Furthermore, we randomly selected six DAPs from the proteome sequencing data and examined the variations in their transcript levels using qRT-PCR. The results showed that the abundance variations of the six candidate proteins under high CO_2_ treatment were consistent with the trend of transcriptional expression (Figure S6).

**Figure 7:**
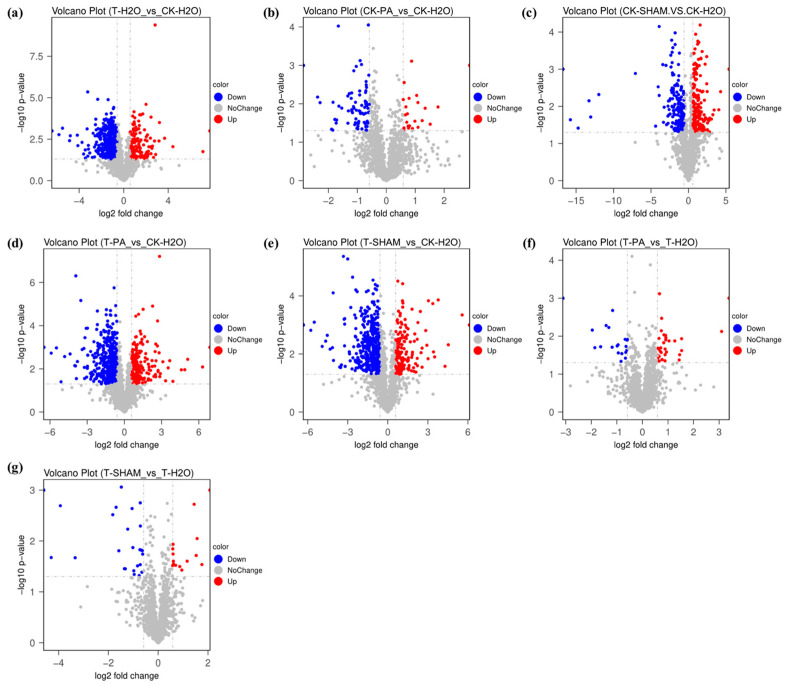
Volcano maps of differentially expressed proteins (DEPs) in *R. pseudoacacia* under CO_2_ treatment. The horizontal coordinate is log2 (fold change), and the vertical coordinate is the negative logarithm of the *p*-value of the *t*-test significance test –log10 (padj). (**a**) T-H_2_O vs. CK-H_2_O; (**b**) CK-PA vs. CK-H_2_O; (**c**) CK-SHAM vs. CK-H_2_O; (**d**) T-PA vs. CK-H_2_O; (**e**) T-SHAM vs. CK-H_2_O; (**f**) T-PA vs. T-H_2_O; and (**g**) T-SHAM vs. T-H_2_O. Red represents upregulated proteins, blue represents downregulated proteins, grey represents proteins with no differential change, and the dotted grey line in the middle represents the threshold line for the DAP screening criteria.

After conducting a one-way ANOVA test, we screened the differential protein data using Fold Change in Expression (FC) ≥ 1.5-fold as a criterion. To investigate the response mechanism of the tetraploid *R. pseudoacacia* under high CO_2_ treatment, we performed a systematic clustering analysis of the differentially abundant proteins (DAPs) (Figure S4). The results revealed a significant change in the expression of DAPs under high CO_2_ treatment. Under normal CO_2_ conditions, in the CK-PA/CK-H_2_O group, we found 50 DAPs, with 11 proteins’ abundances increased and 39 proteins’ abundances decreased (Figure 7b). Similarly, in the CK-SHAM/CK-H_2_O group, we observed 194 DAPs, with 82 proteins’ abundances increased and 112 proteins’ abundances decreased (Figure 7c). In the T-H_2_O/CK-H_2_O group, we identified 504 DAPs, out of which 142 proteins were in elevated abundance, including 3-hydroxybutyryl-CoA dehydrogenase (I3SLI5), glutamine synthetase (I3T8A0), and cytochrome c oxidase subunit Vb (I3SVI7), while 362 proteins were reduced in abundance, including calnexin homolog (I3SD49), glutathione transferase (I3T7D8), and the NAD-dependent epimerase/dehydratase domain-containing protein (I3SMX7) (Figure 7a). In the T-PA/CK-H_2_O group, we detected 522 DAPs, with 351 proteins, including aquaporin PIP13 (A0A0U2BZJ5), photosystem II protein D1 (A0A1C7D3T6), and class V chitinase CHIT5 (A0A1B1J8Z2), showing a significant reduction in abundance. On the other hand, xylose isomerase (I3S153), NADH-ubiquinone oxidoreductase chain 5 (G9JLS7), cytochrome c oxidase subunit 3 (G9JLU6), and 171 other proteins exhibited a significant increase in abundance (Figure 7d). In the T-SHAM/CK-H_2_O group, a total of 506 DAPs were identified. Among these, the abundance of 155 proteins, including ATP synthase β-subunit (Q9FUC8), 3-hydroxybutyryl-CoA dehydrogenase (I3SLI5), and 15-cis-phytoene synthase (I3SHI7), was significantly reduced. On the other hand, the relative abundance of 351 proteins, including peroxidase (I3SDX6), ribonuclease T (I3SNB3), and the prolyl 4-hydroxylase alpha subunit domain-containing protein (I3SZ73), was significantly increased (Figure 7e). In the T-PA/T-H_2_O group, there were a total of 37 DAPs, with 20 proteins’ abundances increased and 17 proteins’ abundances decreased (Figure 7f). Similarly, in the T-SHAM/T-H_2_O group, there were 35 DAPs, with 16 being upregulated and 19 being downregulated (Figure 7g).

Under normal CO_2_ conditions, the comparison of CK-PA/CK-H_2_O and CK-SHAM/CK-H_2_O revealed 26 overlapping DAPs (Figure S3A). However, under high CO_2_ treatment, there was a significant increase in the number of DAPs in the tetraploid *R. pseudoacacia*, with a total of 388 overlapping DAPs in T-PA/CK-H_2_O and T-SHAM/CK-H_2_O (Figure S3B). When comparing T-PA/T-H_2_O and T-SHAM/T-H_2_O to T-H_2_O, there were only 19 overlapping DAPs after PA and SHAM treatments at high CO_2_ concentrations (Figure S3C).

### 2.7. KOG Functional Annotation and GO Enrichment Analysis of Tetraploid R. pseudoacacia DAPs’ Response to High CO_2_

**Figure 8:**
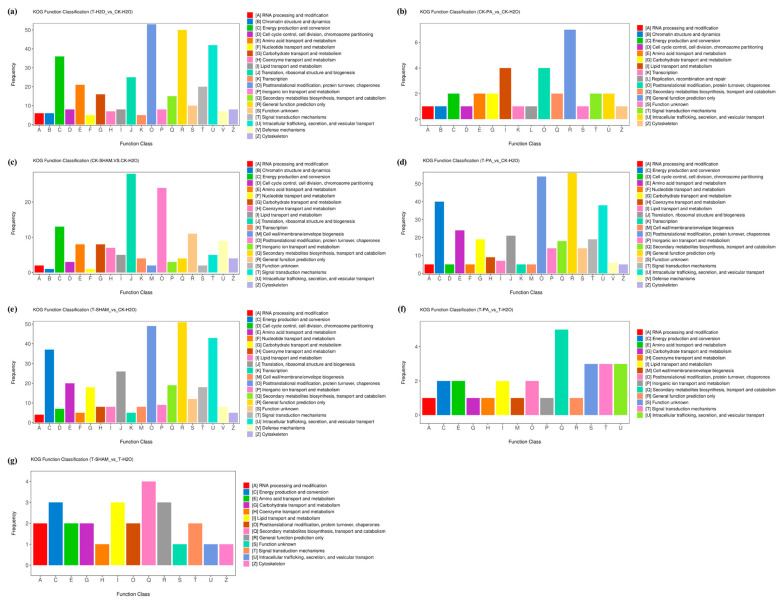
Cluster analysis of orthologous groups (KOG/COG)of DEPs in *R. pseudoacacia* under CO_2_ treatment. (**a**) T-H_2_O vs. CK-H_2_O; (**b**) CK-PA vs. CK-H_2_O; (**c**) CK-SHAM vs. CK-H_2_O; (**d**) T-PA vs. CK-H_2_O; (**e**) T-SHAM vs. CK-H_2_O; (**f**) T-PA vs. T-H_2_O; and (**g**) T-SHAM vs. T-H_2_O.

The distribution of KOG functions was analyzed, and the results showed that, under high CO_2_ treatment, the most abundant functions in T-H_2_O/CK-H_2_O (52 DAPs) were posttranslational modification, protein turnover, and chaperones; nucleotide transport and metabolism, translation, ribosomal structure and biogenesis, and energy production and conversion included 48, 41, and 35 DAPs, respectively (Figure 8a). In the case of normal CO_2_ treatment, the DAPs in CK-PA/CK-H_2_O were mainly associated with general function prediction only, lipid transport and metabolism, posttranslational modification, protein turnover, and chaperones (Figure 8b), whereas the DAPs in CK-SHAM/CK-H_2_O were mainly distributed in translation, ribosomal structure and biogenesis, posttranslational modification, protein turnover, and energy production and conversion (Figure 8c). Under high CO_2_ treatment, the DAPs in T-PA/CK-H_2_O were mainly associated with general function prediction only, posttranslational modification, protein turnover, chaperones, and energy production and conversion (Figure 8d). Similarly, the DAPs in T-SHAM/CK-H_2_O were mainly related to general function prediction only, posttranslational modification, protein turnover, chaperones, intracellular trafficking, secretion, and vesicular transport (Figure 8e). There was no significant difference in the distribution of KOG function between T-PA/T-H_2_O and T-SHAM/T-H_2_O after supplementation with PA and SHAM under high CO_2_ treatment (Figure 8f,g).

In the GO enrichment analysis, DAPs were categorized into three groups: biological processes, molecular functions, and cellular components. Under normal CO_2_ conditions, there were fewer DAPs in the CK-PA/CK-H_2_O group with no significant differential enrichment (Figure S5a). For the CK-SHAM/CK-H_2_O group, the most enriched biological processes were cellular (128 DAPs) and metabolic (118 DAPs) processes; the most enriched molecular functions were catalytic activity (107 DAPs) and binding (99 DAPs); and the most enriched cellular components were cell parts (142 DAPs) and intracellular parts (135 DAPs) (Figure S5c). Under high CO_2_ treatment, the most responsive biological processes were cellular (287 DAPs) and metabolic (252 DAPs) processes in the T-H_2_O/CK-H_2_O group; the most responsive molecular functions were catalytic activity (259 DAPs) and binding (259 DAPs); and the most responsive cellular components were cell parts (327 DAPs) and intracellular parts (309 DAPs) (Figure S5b). The significantly enriched categories of each GO classification in the T-PA/CK-H_2_O and T-SHAM/T-H_2_O groups largely overlapped with those in the T-H_2_O/CK-H_2_O group (Figure S5d,e). These results indicate a significant increase in GO-enriched DAPs under high CO_2_ treatment.

### 2.8. Pathway Analysis of DAPs in Tetraploid R. pseudoacacia under High CO_2_ Treatment

**Figure 9:**
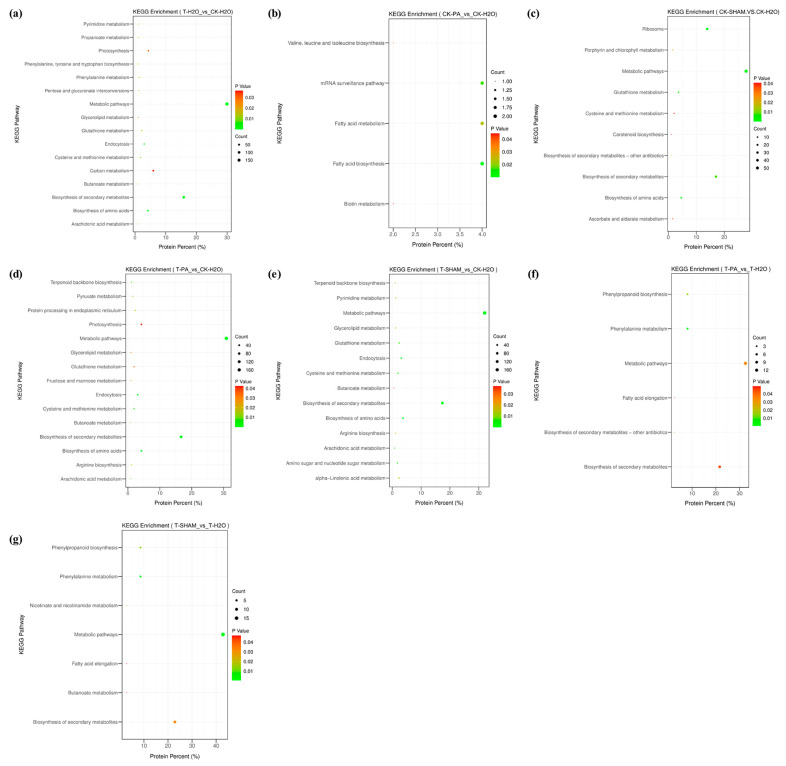
KEGG enrichment analysis of DEPs identified in *R. pseudoacacia* under CO_2_ treatment. (**a**) T-H_2_O vs. CK-H_2_O; (**b**) CK-PA vs. CK-H_2_O; (**c**) CK-SHAM vs. CK-H_2_O; (**d**) T-PA vs. CK-H_2_O; (**e**) T-SHAM vs. CK-H_2_O; (**f**) T-PA vs. T-H_2_O; and (**g**) T-SHAM vs. T-H_2_O. The *x*-axis represents the enrichment factor, and the *y*-axis represents the pathway of enrichment. Larger orange points represent major pathway enrichment and higher pathway impact values, respectively.

To further elucidate the metabolic pathways involved in the DAPs of the tetraploid *R. pseudoacacia*, DAPs were analyzed for KEGG enrichment. Under normal CO_2_ conditions, the DAPs in the CK-PA/CK-H_2_O group were mainly enriched in fatty acid biosynthesis, fatty acid metabolism, and biotin metabolism (Figure 9b). In the CK-SHAM/CK-H_2_O group, the DAPs were mainly enriched in metabolic pathways, the biosynthesis of secondary metabolites, glutathione metabolism, and carotenoid biosynthesis (Figure 9c). Under high CO_2_ treatment, the DAPs in the T-H_2_O/CK-H_2_O group were significantly enriched in metabolic pathways, carbon metabolism, the biosynthesis of secondary metabolites, the biosynthesis of amino acids, and photosynthesis (Figure 9a). The DAPs in the T-PA/CK-H_2_O and T-SHAM/CK-H_2_O groups were significantly enriched in metabolic pathways, the biosynthesis of secondary metabolites, the biosynthesis of amino acids, phenylalanine metabolism, phenylacetone biosynthesis, and glutathione metabolism (Figure 9d,e). Finally, the DAPs in the T-PA/T-H_2_O and T-SHAM/T-H_2_O groups were mainly enriched in phenylalanine metabolism, metabolic pathways, the biosynthesis of secondary metabolites, and phenylpropanoid biosynthesis (Figure 9f,g).

## 3. Discussion

Short-term CO_2_ enrichment has been found to alleviate the impact of abiotic stresses, such as drought and salt, on plants [47,48]. In the tetraploid *R. pseudoacacia*, prolonged exposure to high CO_2_ resulted in a gradual decrease in the relative water content of its leaves over time, ultimately leading to the inhibition of its growth and development. In the present study, we observed the yellowing, wilting, and abscission of leaves, as well as a decrease in the relative water content in the tetraploid *R. pseudoacacia* at high CO_2_ concentrations (Figure 1a). This phenomenon could be attributed to a reduction in water flux caused by high CO_2_ and weakened upward water transport due to a decreased transpiration rate [49,50]. These factors contribute to the recovery of plants once the CO_2_ concentration is restored (Figure 1c). The magnitude of the REC can indicate the condition of the plant membrane system. When plants face adversity or injuries, the cell membrane may rupture, causing a leakage of cytoplasmic cytosol and consequently increasing the conductivity. The substantial increase in REC observed at high CO_2_ concentrations suggests that the plant experienced stress injury and cell damage (Figure 1b). PA, known as a facilitator of the alternate respiration pathway, and SHAM, an inhibitor [32,33,35], have shown contrasting effects on the stress symptoms of the tetraploid *R. pseudoacacia*. PA supplementation alleviated the stress response of the tetraploid *R. pseudoacacia* to high CO_2_, whereas SHAM supplementation exacerbated this stress response (Figure 1).

Photosynthesis involves important physiological indicators used to study the impact of abiotic stress on plants. High levels of CO_2_ can reduce the photosynthetic capacity of plants. This reduction is mainly seen through decreased CO_2_ assimilation capacity and photochemical activities [10,13,51]. In our study, we observed a decrease in photosynthetic parameters in the tetraploid *R. pseudoacacia*’s leaves when exposed to high CO_2_, indicating the inhibition of photosynthetic performance. However, after treatment with PA, the photosynthetic performance showed signs of recovery. After SHAM treatment, photosynthesis in the tetraploid *R. pseudoacacia* was found to be even more inhibited (Figure 1e–g). This could be due to a decrease in photosynthetic parameters resulting from a reduction in the amount and activity of Rubisco, stomatal conductance, and photorespiration [52,53].

A high concentration of CO_2_ induces an accumulation of ROS in plants [54,55]. Previous studies have shown that an excessive accumulation of electrons in the PSII leads to an over-reduction in electrons, which in turn causes the electron transport chain to become highly reduced, resulting in the formation of ROS. This oxidative stress not only causes damage to plants but also triggers retrograde signals to the nucleus, leading to responses at the gene expression level [56,57]. In the tetraploid *R. pseudoacacia*, there was a significant increase in ROS levels compared to the control group under high CO_2_. This change in ROS levels was observed when the AOX pathway was stimulated or inhibited (Figure 2a–c), which aligns with Dinakar’s study [58]. Algae have developed various antioxidant defense mechanisms to mitigate the oxidative stress caused by H_2_O_2_. These mechanisms include enzymatic processes involving the ascorbate–glutathione cycle, glutathione S-transferase (GST), glutathione peroxidase (GPX), and CAT. Additionally, nonenzymatic mechanisms related to carotenoids and glutathione also play a role [59,60,61]. Most ROS-scavenging enzymes, such as CAT, SOD, and APX, are predominantly bound to cystoid membranes and exhibit similarities. When plants experience stress, they promptly remove ROS to prevent toxicity caused by ROS diffusion into the matrix [61]. To maintain ROS homeostasis, the activity of antioxidant enzymes is typically enhanced to eliminate excess ROS, thus safeguarding the plant from oxidative damage and enhancing its stress tolerance [62,63]. However, this mechanism is effective against mild oxidative damage, and if the damage surpasses the plant’s tolerance threshold, the inability to eliminate ROS will hinder the functioning of antioxidant enzymes [64,65]. In this study, high concentrations of CO_2_ led to a significant increase in ROS levels in the tetraploid *R. pseudoacacia*, exceeding the capacity of the antioxidant system and resulting in the suppression of SOD, CAT, GR, and APX activities, ultimately causing damage to the plant (Figure 2d–f).

Chlorophyll content can have a direct impact on a plant’s capacity to capture light energy. The decrease in chlorophyll content can be attributed to two factors: the blockage of chlorophyll synthesis and the degradation of chlorophyll [66]. In our study, we observed that the levels of both Chl a and b initially increased and then decreased under high CO_2_ treatments (Figure 3a,b). This finding aligns with previous research and suggests that high concentrations of CO_2_ may inhibit chlorophyll synthesis [67]. We investigated the impact of an elevated CO_2_ concentration on the activity of PSII using the chlorophyll fluorescence technique. The intensity of chlorophyll fluorescence is an indicator of the redox state of the main receptor Q in PSII [68]. Our findings revealed that the treatment with high CO_2_ levels resulted in an increase in *Fo* but a significant decrease in Fm and *Fv/Fm* in the tetraploid *R. pseudoacacia* (Figure 3d–f). This suggests that the activity of PSII was hindered, leading to an excessive accumulation of electrons in PSII. Additionally, *q*P and NPQ represent the capacities for photochemical and nonphotochemical excitation quenching, respectively, and the quantum yields of these two types of fluorescence quenching are interconnected [69]. A decrease in *ETR* signifies a decline in photosynthetic activity. In the tetraploid *R. pseudoacacia*, photosynthesis is hindered, disrupting the balance of the system. When the plant is under stress, PSII is inhibited (Figure 3), resulting in the generation of a chlorophyll triplet state and singlet oxygen (^1^O_2_), which ultimately leads to the accumulation of ROS.

The regulation of the mitochondrial electron transport chain, which includes the cytochrome respiratory (COX) and AOX respiratory pathways, is influenced by abiotic stress. Under high CO_2_ conditions, AOX respiration is upregulated in plant mitochondria to support normal plant physiological activities [70]. Research has demonstrated the significance of the AOX pathway in *A. thaliana* in response to elevated CO_2_ [71]. It was observed that the rate of cytochrome respiration in the tetraploid *R. pseudoacacia* decreased, while the rate of AOX respiration increased under high CO_2_ stress. However, overall respiration was inhibited. AOX respiration, which is a SHAM-sensitive type of respiration, was stimulated by PA treatment and had a significant effect on AOX [32,33,35]. PA treatment notably enhanced the AOX respiration pathway, while the response of AOX respiration in the tetraploid *R. pseudoacacia* to high CO_2_ was inhibited by SHAM (Figure 4d). In contrast, the mitochondrial transfer chain (ETC) complexes (I, II, III, and IV) exhibited distinct responses under high CO_2_. The activities of complexes I and II were decreased, while the activities of complexes III and IV were downregulated (Figure 4e–h). This alteration in activity levels could be attributed to mitochondrial damage resulting from the accumulation of ROS.

Low CO_2_ promotes stomatal opening, while high CO_2_ induces stomatal closure. Stomata, which serve as the primary site for gas exchange, can adjust the pore size in response to changes in the external environment. Studies have shown that high CO_2_ stimulates an increase in the concentration of free Ca^2+^ in guard cells, leading to the induction of stomatal closure [14,15,16,17]. Transient changes in the pH and membrane potential of guard cells occur alongside stomatal closure when the CO_2_ concentration is increased [72]. Stomata control the size of pores in the leaf in response to CO_2_ concentration, and elevated CO_2_ also inhibits stomatal development [73,74]. Previous studies have demonstrated that high levels of CO_2_ can induce stomatal closure, with ROS playing a crucial role in this regulatory process [22,75]. NO plays a role in both ABA- and Ca^2+^-mediated stomatal closure, which is an important participant in the stomatal closure process [24,25,76]. When compared with normal CO_2_, high CO_2_ significantly induces stomatal closure. PA alleviates this process, while SHAM exacerbates the degree of closure. Additionally, SHAM also causes a slight closure of stomata at normal CO_2_ concentrations. Through fluorescence staining, it was observed that CO_2_-induced stomatal closure is accompanied by a large accumulation of H_2_O_2_ and NO in the guard cells (Figure 5 and Figure 6). This provides evidence for the crucial role of ROS and NO in the stomatal closure of the tetraploid *R. pseudoacacia* in response to high CO_2_ stress.

Quantitative proteomics analysis is a crucial technology in proteomics research [77]. We employed DIA quantitative proteomic analysis to investigate protein alterations in the tetraploid *R. pseudoacacia* under high CO_2_ treatment. The identified proteins were found to be involved in significant pathways, including metabolic pathways, carbon metabolism, the biosynthesis of secondary metabolites, the biosynthesis of amino acids, and photosynthesis (Figure 7, Figure 8 and Figure 9). Additionally, we screened several key proteins in all DAPs in response to high CO_2_, including ribulose diphosphate carboxylase-related subunits (A0A0F6Y5S8, C0J370), photosystems I-II-related proteins (A0A898CWR3, A0A898CTK1, A0A898CW46, A0A898CTN3, A0A898CW14), antioxidant-related proteins (I1KP94, A0A0R4J3P1, K7KDM7, A0A0R0ERX9), cytochrome-related proteins (A0A898CTT0, I1MWP7, A0A0R0H975), and NADPH-related proteins (A0A0R4J3V9, I1LT63) (Table S2).

In C3 plants, glucose is primarily biosynthesized through the Calvin cycle. A crucial step in this process is the formation of glycerate-3-phosphate (3PGA) through the action of 1,5-bisphosphate ribulose carboxylase using CO_2_ [28]. Rubiscos, which are photosynthetic CO_2_-fixing enzymes, are activated by Rubisco activase (Rcas). However, if the N-terminus of the large subunit is missing, Rubiscos are not activated [78]. The involvement of carbon dioxide is also essential for the activation of Rubiscos [79]. In this study, we observed a significant downregulation of many 1,5-bisphosphate ribulose carboxylase-related subunits (I6QMJ2, A0A0F6Y5S8, C0J370, A0A898CTM2) under high CO_2_ treatment. This downregulation resulted in the inability of Rubiscos to be activated under light and CO_2_ conditions, leading to a decrease in the photosynthesis rate. Furthermore, the inhibition of photosynthesis was also attributed to the impact on photosystem protein synthesis, electron transfer between photosystems, and chlorophyll binding [80,81,82]. Pigment–protein complexes CP43 and CP47 are known to transfer excitation energy from the outer antenna of photosystem II to the photochemical reaction center, as well as being involved in the process of water oxidation [83,84]. The excess energy absorbed by the photosystem I complex is transferred to P700 via chlorophyll, thus protecting the pigment–protein complex from photodestruction [85]. The photosystem II D1/D2 complex proteins catalyze the oxidation of water due to a high REDOX potential [86]. Light trapping and the conversion of light energy to chemical energy occur through the iron–sulfur center in photosystem I [87,88]. In our study, we observed a significant downregulation of various photosystem constitutive proteins in the tetraploid *R. pseudoacacia*. These proteins include photosystem II CP43/47 reaction center proteins (A0A6H0EHP1, A0A1C7D4D5), photosystem I P700 chlorophyll a apoprotein A1/A2 (A0A1C7D3U7, A0A1C7D4C6, A0A6H0EHR0), photosystem I reaction center subunit II (I3STB2), photosystem II D1/D2 proteins (A0A1C7D3T6, A0A1C7D3V4), electron-transfer-mediating protein cytochrome f (A0A1C7D3W5), plastoquinone–plastocyanin reductase (I3RZ73, I1LUB3), the photosystem I iron–sulfur center (A0A1C7D3Z6), and the chlorophyll a–b binding protein (I3SIW2). This downregulation resulted in the inhibition of electron transfer and light capture, ultimately leading to the blockage of photosynthesis in the tetraploid *R. pseudoacacia*.

Glutathione (GSH) plays a crucial role in various biological processes. When GSH is oxidized to oxidized glutathione (GSSG) by GPX, it catalyzes the reduction of H_2_O_2_. Additionally, GR helps in regenerating GSH. Additionally, GSH has been found to effectively increase the antioxidant capacity of plants and enhance the activity of respiratory enzymes [89]. In certain studies, advanced GSH treatment has proven to be effective in preventing broccoli fermentation in a high CO_2_ atmosphere. This is achieved by promoting the AsA–GSH cycle and the electron (ETC) pathway [78]. Exogenous PA can maintain intracellular glutathione levels in H_2_O_2_-treated cells and enhance their antioxidant capacity [90]. The presence of GSH is vital for copepods in defending against seawater acidification induced by varying CO_2_ concentrations [91]. In this study, the expression of GPX (I3SK85, I1KP94, I1MX60, C6SY48) was significantly decreased by high CO_2_ treatment. However, this decrease was reversed when PA was applied, indicating that the antioxidant capacity of the tetraploid *R. pseudoacacia* was restored. The decrease in GPX expression resulted in the accumulation of ROS. GR plays a crucial role in the interconversion between oxidized and reduced glutathione, facilitating the recirculation of GSH. In soybeans, enhancing GR can effectively improve the removal of ROS [92]. In the context of this experiment, high CO_2_ levels led to the downregulation of GR and diminished the regeneration ability of GSH. However, the application of PA facilitated the conversion between GSH and GSSG under high CO_2_ conditions, which enhanced the scavenging of H_2_O_2_ and alleviated oxidative damage.

## 4. Materials and Methods

### 4.1. Plant Materials and Stress Treatment

The plant materials comprised the tetraploid *R. pseudoacacia*, which was two years old. In the spring, bare-root seedlings were planted in uniform-sized pots (diameter × height × bottom diameter = 20 × 28 × 20 cm), and seedlings with uniform growth were chosen for CO_2_ treatment after 2 months of growth under normal conditions. The potted seedlings were placed in a light incubator for 3 days for pre-cultivation to acclimate to the new environment. The soil moisture was maintained at 70% throughout, and the culture conditions were as follows: 16 h of light, 8 h of darkness, temperature set at 25 °C, air humidity at 70%, and light intensity at 6000 Lux. The seedlings undergoing the high CO_2_ treatment were divided into a control group (natural conditions, a CO_2_ concentration of about 0.031%) and treatment groups (a CO_2_ concentration of 5%), and the seedlings were subjected to high CO_2_ treatment for 9 days before being incubated again under normal CO_2_ concentration for 3 days. On day 6 of CO_2_ treatment, a respiration accelerator (pyruvic acid, 0.1 mM) and a respiration inhibitor (salicylhydroxamic acid, 1 mM) were sprayed on the leaves of the tetraploid *R. pseudoacacia* every 3 h. Morphological observations on leaves were made after 0 d, 3 d, 6 d, 9 d, and 12 d. For each treatment, leaves were collected for physiological and proteomic measurements, with three or six biological replicates.

Here, CK-H_2_O: untreated control group; CK-PA: pyruvic acid-treated group; CK-SHAM: salicylhydroxamic acid-treated group; T-H_2_O: CO_2_-treated group; T-PA: CO_2_ co-treated with PA; and T-SHAM: CO_2_ co-treated with SHAM.

### 4.2. Determination of Relative Water Content and Relative Electrical Conductivity

After 0.5 g of fresh leaves (FWs) moistened in distilled water for 4 h, the surface water was removed, weighed, and recorded as TW, and then the leaves were dried in a drying oven and recorded as DW. Relative water content: RWC (%) = (FW − DW)/(TW − DW) × 100.

A total of 0.1 g of evenly chopped leaves were poured in 5 mL of ddH_2_O and shaken at 160 rpm for 1 h. The electrical conductivity was measured and recorded as L2 using a DDS-IIA conductivity meter (INESA Scientific Instruments Co., Shanghai, China), and the control of ddH_2_O was recorded as L1. After cooling to ambient temperature, the samples were immersed in a boiling water bath for 15 min, and the electrical conductivity was measured and recorded as L3. Relative electrical conductivity (%) = (L2 − L1)/(L3 − L1) × 100.

### 4.3. Determination of Chlorophyll Content and Photosynthetic and Fluorescence Parameters

The chlorophyll content of the fourth and fifth leaves from the end, located in the middle of the acacia branches, was determined. A total of 0.2 g of leaves were immersed in the extraction solution (80% acetone/ethanol/ddH_2_O = 4.5:4.5:1). The leaves were then kept in the dark for 24 h. The optical density at wavelengths of 470 nm, 645 nm, and 663 nm was measured. Chlorophyll a (Chl a) = 12.72A_663_ − 2.59A_645_; chlorophyll b (Chl b) = 22.88A_645_ − 4.67A_663_; and Chl a/b = chlorophyll a/b.

The photosynthetic parameters of leaves were determined using a Li-COR 6400 photosynthesis system (LI-COR, Lincoln, NE, USA). Six leaves per group were analyzed, and the measurements were repeated three times. These parameters included the net photosynthetic rate (*P*n), the transpiration rate (*T*r), the intercellular carbon dioxide concentration (*C*i), and the stomatal conductance (*G*s). The measurements were conducted under specific conditions, including a temperature of 24 °C, a photon flux density (PFD) of 90 µmol m^−2^·s^−1^, a relative humidity of 70%, and an ambient CO_2_ concentration of 400 µmol CO_2_ mol^−1^.

The chlorophyll fluorescence parameters of leaves were measured using an FC800-O fluorescence imaging system (FluorCam, Drásov, Czech Republic) [93]. The leaves were dark-adapted for 30 min, following the previously described method. The data collected included initial fluorescence yield (*Fo*), maximum fluorescence yield (*Fm*), photochemical quenching (*q*P), the maximum photochemical efficiency of photosystem II (PSII) (*Fv/Fm*), nonphotochemical quenching (NPQ), and the relative electron transfer rate (ETR). A total of six leaves were measured for each treatment group.

### 4.4. Determination of Leaf Respiration Parameters

The leaf respiration rate was determined using the OXYTHE-RM oxygen electrode. The total respiration rate (V_t_) was determined by incubating 0.1 g leaves of *R. pseudoacacia* from the same location in a reaction medium containing 2 mM HEPES, 10 mM MES (pH 10.7), and 2 mM CaCl_2_ for 30 min in the dark. To determine the residual respiration (V_res_), SHAM (200 mM) and KCN (200 mM) were added to the assay. V_res_ was then subtracted from the respiratory activity observed when SHAM was added alone to obtain the activity of the major cytochrome pathway (V_cyt_). Similarly, V_t_ was subtracted from the respiratory activity observed when SHAM was added alone to obtain the actual activity of the alternative pathway (V_alt_).

The activity of mitochondrial electron transport chain complex I-IV (complex I-IV) and AOX was measured in the central leaves of *R. pseudoacacia* using the Plant Mitochondrial Respiratory Chain Complex I-IV and Plant AOX Activity Kit (Hengyuan Biologicals, Shanghai, China).

### 4.5. Stomatal Movement

Under controlled light and temperature conditions, fresh leaves were collected from plants grown under normal conditions and with high CO_2_ treatments. The upper epidermis and leaf pulp of both the proximal and distal axes of the leaf blades were gently scraped. Stomatal morphology was then observed randomly using a microscope. More than 100 stomata were selected for each treatment sample. The length and width of the stomatal images were measured using ImageJ v1.8.0 to calculate the stomatal openness.

### 4.6. Fluorescent Probe Staining

Fresh leaves were collected from plants under both normal conditions and high CO_2_ treatments after 0, 6, and 12 days. Leaf blades, excluding the main veins, were cut into 1 cm pieces. These pieces were then immersed in 50 mM H_2_DCF-DA or 0.1 mM DAF-2DA for 30 min in the absence of light. Subsequently, they were photographed using a fluorescence microscope in 10 randomly selected fields of view. The average fluorescence intensity was calculated using ImageJ.

### 4.7. Histochemical Staining

The leaves were fully immersed in a staining solution containing 10 mg·mL^−1^ DAB and NBT. To eliminate air bubbles, a vacuum pump was used for 30 min. Subsequently, the leaves were stained under dark conditions for 24 h. Chlorophyll was completely removed by submerging the leaves in a decolorizing solution. The leaves were then photographed and recorded, and the average fluorescence intensity was measured using ImageJ.

### 4.8. Determination of H_2_O_2_ and O_2_^·−^ Content

A total of 0.5 g of leaves were homogenized in 0.1% TCA in an ice bath. The homogenate was then centrifuged at 12,000 rpm for 10 min. The extract was mixed with phosphate buffer solution (PBS) (pH 7.5) and KI (1 M). The absorbance of the mixture was measured at 390 nm.

Similarly, a total of 0.5 g of leaves were homogenized in hydroxylamine hydrochloride in an ice bath and then centrifuged at 12,000 rpm for 10 min. The supernatant obtained was incubated with 1-Naphthylamine and aminobenzenesulfonic acid for 2 min at room temperature. Subsequently, the absorbance of the solution was measured at 520 nm [94].

### 4.9. Determination of Antioxidant Enzyme Activity

Crude enzyme extract was prepared by grinding 0.5 g of leaves in liquid nitrogen and centrifuging in PBS at 12,000 rpm for 10 min. A mixture of 20 μL of extract, 50 mM PBS (pH 7.8), 100 μM ethylene diamine tetraacetic acid (EDTA), and 10 mM pyrogallic acid was thoroughly mixed. Superoxide dismutase (SOD) activity was measured by UV spectrophotometry at 420 nm. Another mixture of 0.2 mL of extract, 50 mM PBS, and ddH_2_O was thoroughly mixed, and the reaction was carried out in a water bath at 25 °C for 10 min; catalase (CAT) activity was measured at 240 nm. Additionally, 0.05 mL of extract, PBS, and 5 mM ASA were mixed thoroughly. This mixture was then followed by the addition of 0.1 mM H_2_O_2_, and the activity of ascorbate peroxidase (APX) was measured at 290 nm. Furthermore, a mixture of 10 μL of extract, 100 mM PBS, 2 mM EDTA, and 0.5 mM glutathione was mixed thoroughly. This mixture was then followed by the addition of 0.2 mM NADPH, and the activity of glutathione reductase (GR) was detected at 340 nm [45].

### 4.10. Quantitative Proteomics Analysis

Six sets of samples were pretreated using the iST Sample Pretreatment Kit (PreOmics, Planegg, Germany) and stored at −80 °C under vacuum conditions. The samples were crushed in liquid nitrogen and then mixed with buffer (1:10) and protease inhibitors. After vortexing for 10 min, the samples were vortexed for another 10 min with an equal volume of Tris-saturated phenol (pH 8.0). Subsequently, they were centrifuged at 12,000 rpm for 20 min at 4 °C, and the phenol phase was combined with buffer and vortexed before centrifugation. The precipitate was then treated with pre-cooled ammonium acetate–methanol solution, precipitated overnight at −20 °C, and finally centrifuged at 12,000 rpm for 20 min at 4 °C. The supernatant was discarded, and the precipitate was washed twice with 90% acetone. The precipitate was then suspended in an appropriate volume of lysate to solubilize the sample proteins, followed by centrifugation at 12,000 rpm for 20 min at 4 °C to collect the supernatant. This step was repeated to ensure maximum collection of the supernatant. Each sample was then suspended in 30 μL of solvent A, and 1 μL of 10×iTR peptide was added to 9 μL of each sample. After thorough mixing, the samples were subjected to nanoliquid chromatography. For tandem mass spectrometry analysis, 4 μL of the samples were taken. The separation of the sample occurred over a 90 min gradient with a column flow rate of 600 nL·min^−1^ and a column temperature of 55 °C. The gradient started at 4% B-phase and was equilibrated for 4 min, followed by a nonlinear gradient increase to 30% over 80 min. Subsequently, the gradient increased to 90% in 2 min and was held for 8 min.

The mass spectrometry conditions were as follows: (1) MS: scan range (*m*/*z*): 350–1500; resolution: 120,000; normalized AGC target: 100%; and maximum injection time: 50 ms; (2) HCD-MS/MS: resolution: 30,000; normalized AGC target: 600%; maximum injection time: 90 ms; and collision energy: 35; (3) variable window acquisition was used with 60 windows set up, and overlapping serial ports were set up with 1 m·z^−1^ overlap per window.

Data analysis: DIA data were analyzed using Spectronaut 15.0 default parameters (Omicsolution Co., Ltd., Shanghai, China). The sequence database used was the uniprot-robinioid clade database, and trypsin enzymatic digestion was applied. The search library parameters included a fixed modification, carbamidomethyl (C), and a variable modification, methionine oxidation. The criteria for protein characterization were a precursor threshold of 1.0% false discovery rate (FDR) and a protein threshold of a 1.0% FDR. To generate the decoy database, a mutation strategy was employed, similar to perturbing a random number of amino acid sequences. Spectronaut 15.0 performed auto-correction, and a local normalization strategy was implemented for data normalization. Protein group quantification was conducted by calculating the average of the peak areas of the first 3 peptides below 1.0% FDR. For differential screening, proteins with a *p* adj value < 0.05 and |fold change| > 1.5 were considered significant after analysis by a one-way ANOVA test.

### 4.11. Functional Annotation and Cluster Analysis

An unsupervised principal component analysis (PCA) was conducted on the entire dataset to provide a comprehensive overview of the quantitative proteomics data, assessing its relevance and reproducibility. Volcano plots were then analyzed using a one-way ANOVA test, with a significance threshold of a *p* adj value < 0.05. To identify differentially abundant proteins (DAPs), we considered a fold change greater than 1.5. For the functional annotation and enrichment analysis of the DAPs, we utilized the GO (Gene Ontology), KOG (Eukaryotic Orthologous Groups), and KEGG (Kyoto Encyclopedia of Genes and Genomes) databases, which helped us understand the functions, distributions, and pathways associated with these DAPs.

### 4.12. Data Submission

MS data were translated to PRIDE XML using the PRIDE Submission Tool Version 2.7.3. A total of 34 PRIDE XML files were submitted to the ProteomeXchange repository following the ProteomeXchange submission guidelines [95]. The data were deposited under the identifier PXD047363.

### 4.13. Quantitative Real-Time Polymerase Chain Reaction (qRT-PCR)

Samples were ground to powder in liquid nitrogen, and total RNA was isolated using the Omega Plant RNA Kit (Shanghai Yuanmu Biotechnology Co., Shanghai, China) following the manufacturer’s instructions. cDNA was prepared with the PrimeScript Reverse Transcriptase Kit (Takara, Shiga, Japan). qPCR was performed in a LightCycler 480 System (Roche, Indianapolis, IN, USA) with LightCycler 480 SYBR Green I Master Mix and specific primers (Supplementary Table S1). Three biological replicates were used for each sample. The *18SRNA* gene was used as an internal control. Relative gene expression levels were calculated using the ΔΔC_t_ method after normalization to the reference gene *18SRNA*.

### 4.14. Statistical Analysis

All experiments were performed with at least three to six biological replicates; experimental data were analyzed with SPSS 25.0; and to determine statistical significance, asterisks indicated significant differences (Student’s *t*-test; * *p* ≤ 0.05; ** *p* ≤ 0.01; *** *p* ≤ 0.001).

## 5. Conclusions

In conclusion, this study investigated the response mechanism of the tetraploid *R. pseudoacacia* to high CO_2_ concentrations through physiology and proteomics. The findings indicate that high CO_2_ had a negative impact on the normal growth of the tetraploid *R. pseudoacacia*. Specifically, the photosynthesis and respiration of the tetraploid *R. pseudoacacia*’s leaves decreased under the high CO_2_ treatment compared to normal conditions, leading to an excessive accumulation of ROS. Additionally, the activities of the antioxidant enzymes SOD, CAT, GR, and APX were reduced, and the levels of chlorophyll and chlorophyll fluorescence parameters were significantly diminished. Furthermore, the accumulation of ROS and NO in guard cells induced stomatal closure. The proteomic analysis revealed that the DAPs were primarily enriched in metabolic pathways, the biosynthesis of secondary metabolism, and amino acid biosynthesis pathways under high CO_2_ treatment. Notably, the key DAPs were primarily associated with ribulose diphosphate carboxylase-related subunits, photosystem I-II-related proteins, antioxidant-related proteins, and cytochrome-related proteins. Overall, this study provides valuable insights into the adaptation of the tetraploid *R. pseudoacacia* to high CO_2_ environments. However, further research is required to validate the target proteins.

## Data Availability

MS data were translated to PRIDE XML using the PRIDE Submission Tool Version 2.7.3. A total of 34 PRIDE XML files were submitted to the ProteomeXchange repository following the ProteomeXchange submission guidelines. The data were deposited under the identifier PXD047363.

## Supplementary Materials

The supporting information can be downloaded at https://www.mdpi.com/article/10.3390/ijms25105262/s1.
