# Supplementary material for: Physiological and Proteomic Responses of the Tetraploid Robinia pseudoacacia L. to High CO2 Levels
Source: Int J Mol Sci. 2024 May 11;25(10):5262. doi: 10.3390/ijms25105262 (PMC11121411; doi:10.3390/ijms25105262)
Supplement: Supplementary file 1 [file ijms-25-05262-s001.zip › Supplementary Figures.pdf]

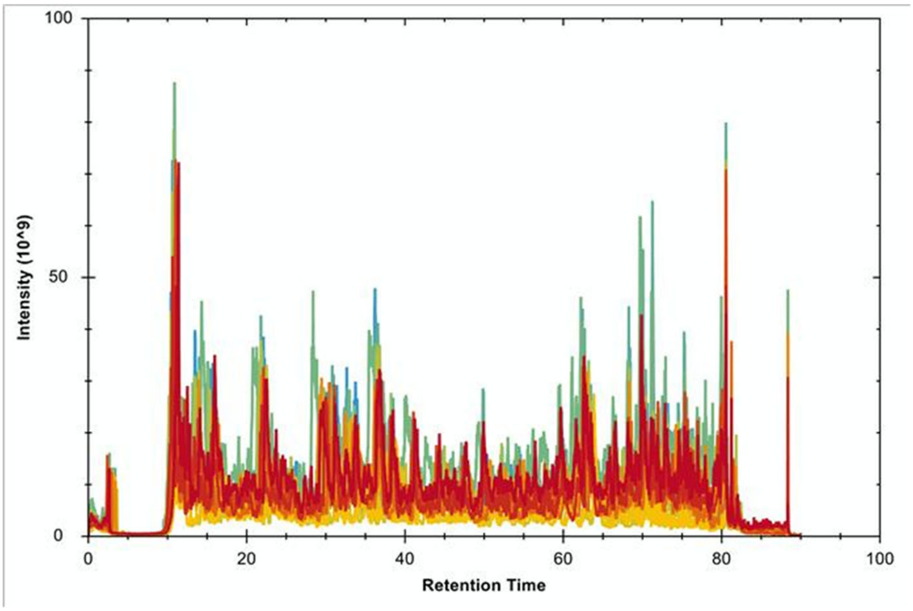

Supplementary Figure 1. Total ion chromatogram of tetraploid *Robinia pseudoacacia*

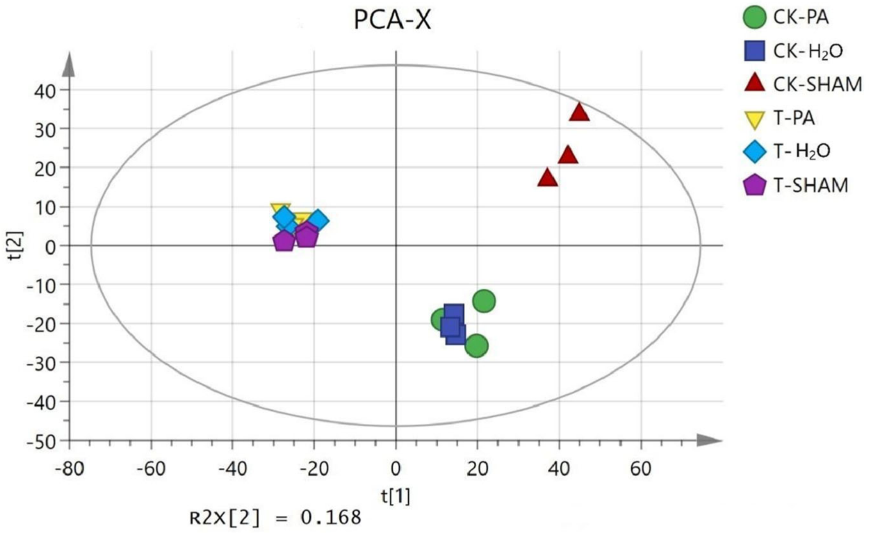

Supplementary Figure 2. Principal Component Analysis (PCA) score plots of the protein profiles of leaves in *Robinia pseudoacacia*. The experiment was carried out with three biological replicates.

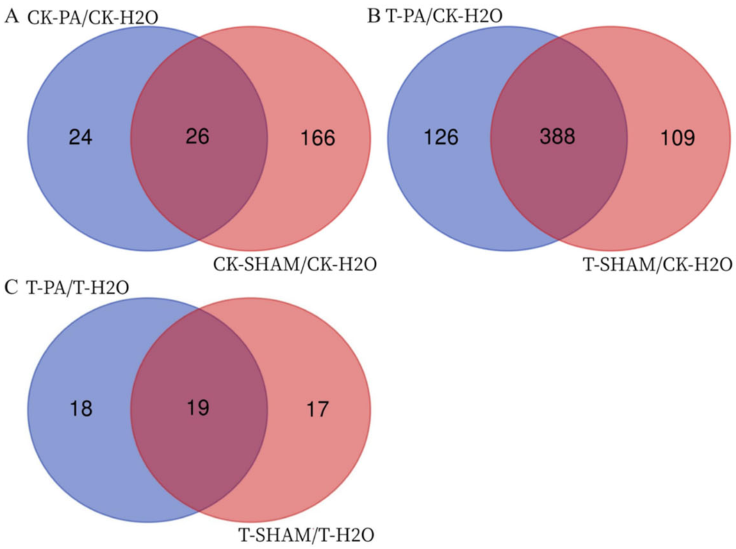

Supplementary Figure 3. Venn diagrams of DAPs in *Robinia pseudoacacia* under high CO_2.

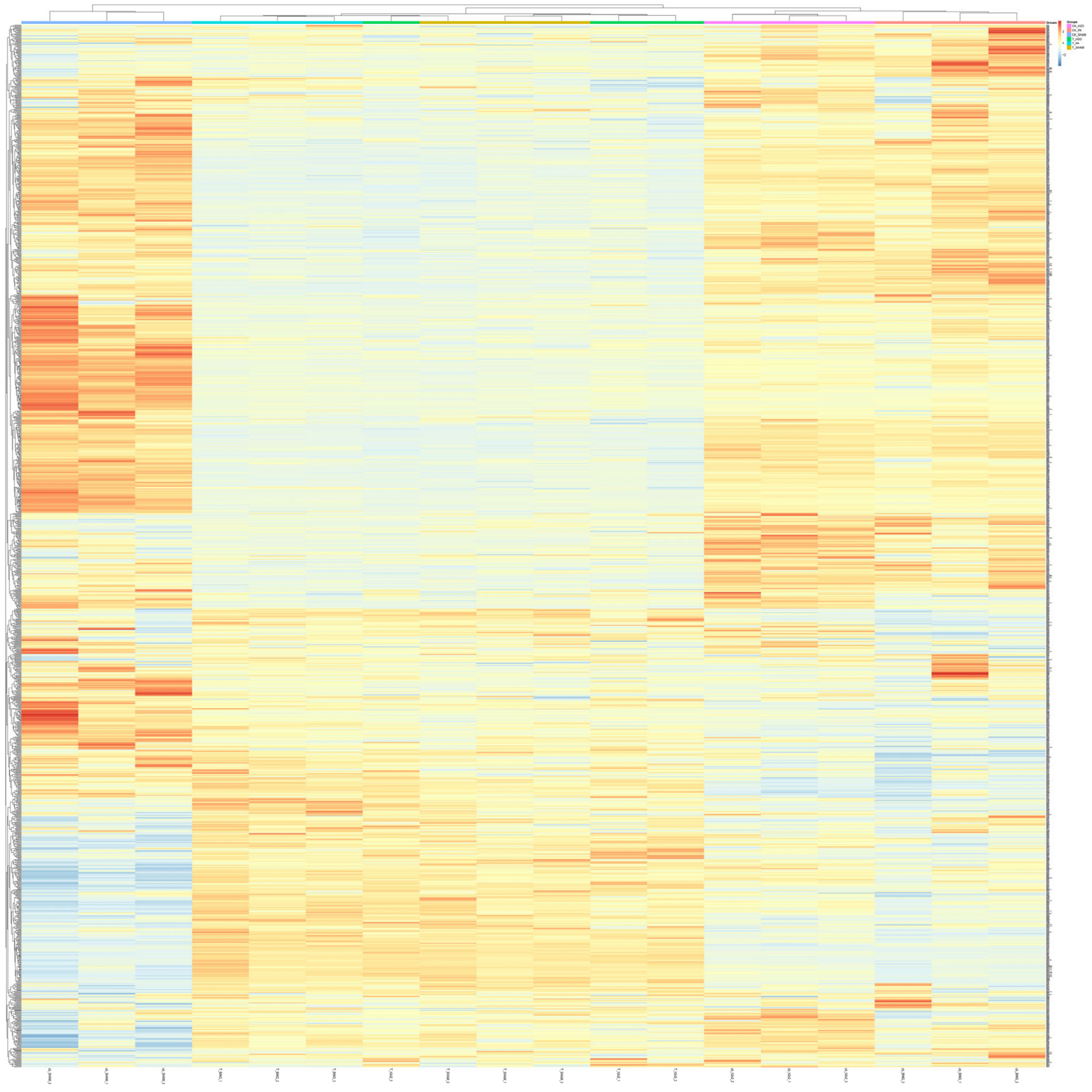

Supplementary Figure 4. Hierarchical Cluster Analysis DEPs in *Robinia pseudoacacia* at high CO_2. The color shades represent the abundance of the proteins.

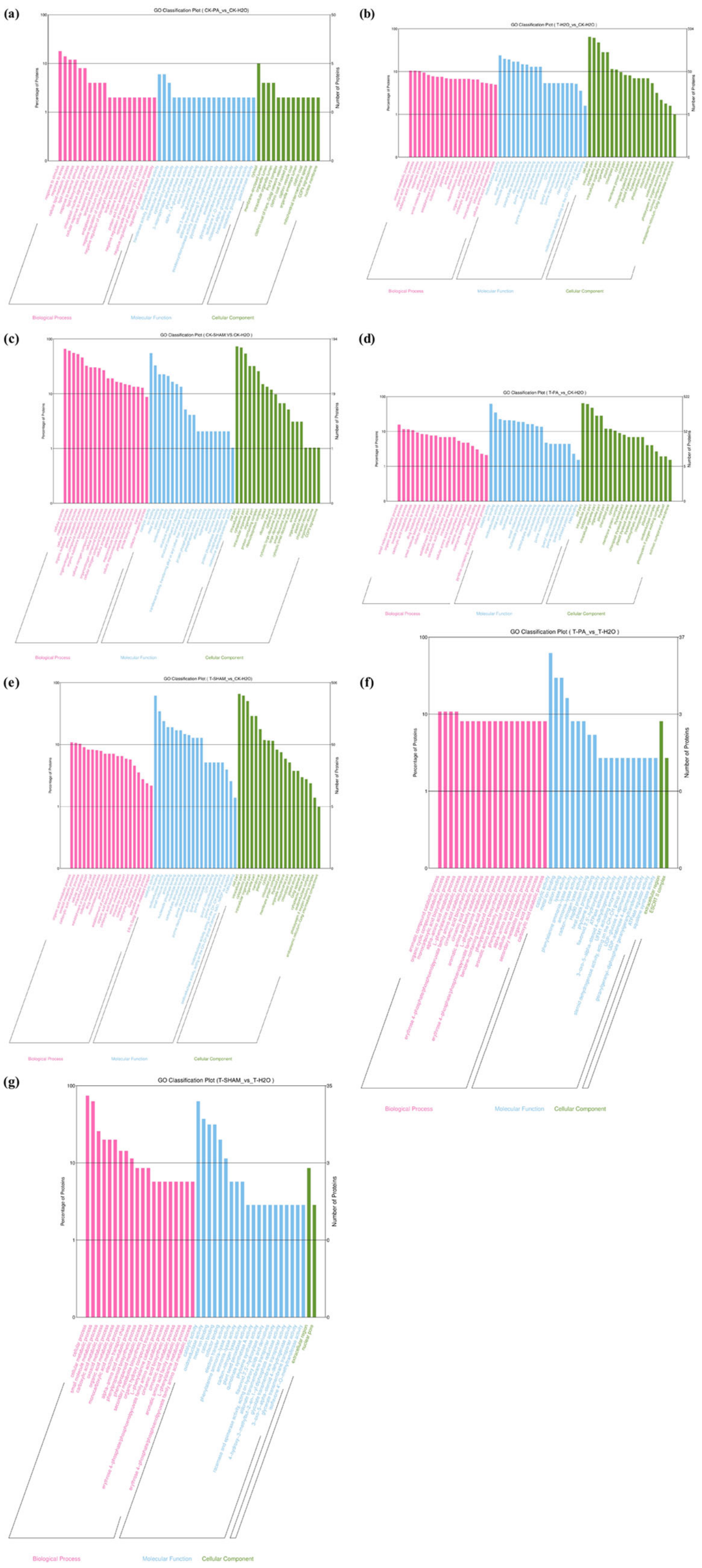

Supplementary Figure 5. GO enrichment clusters of DAPs in *Robinia pseudoacacia* at high CO_2. The color shades represent the abundance of the proteins.

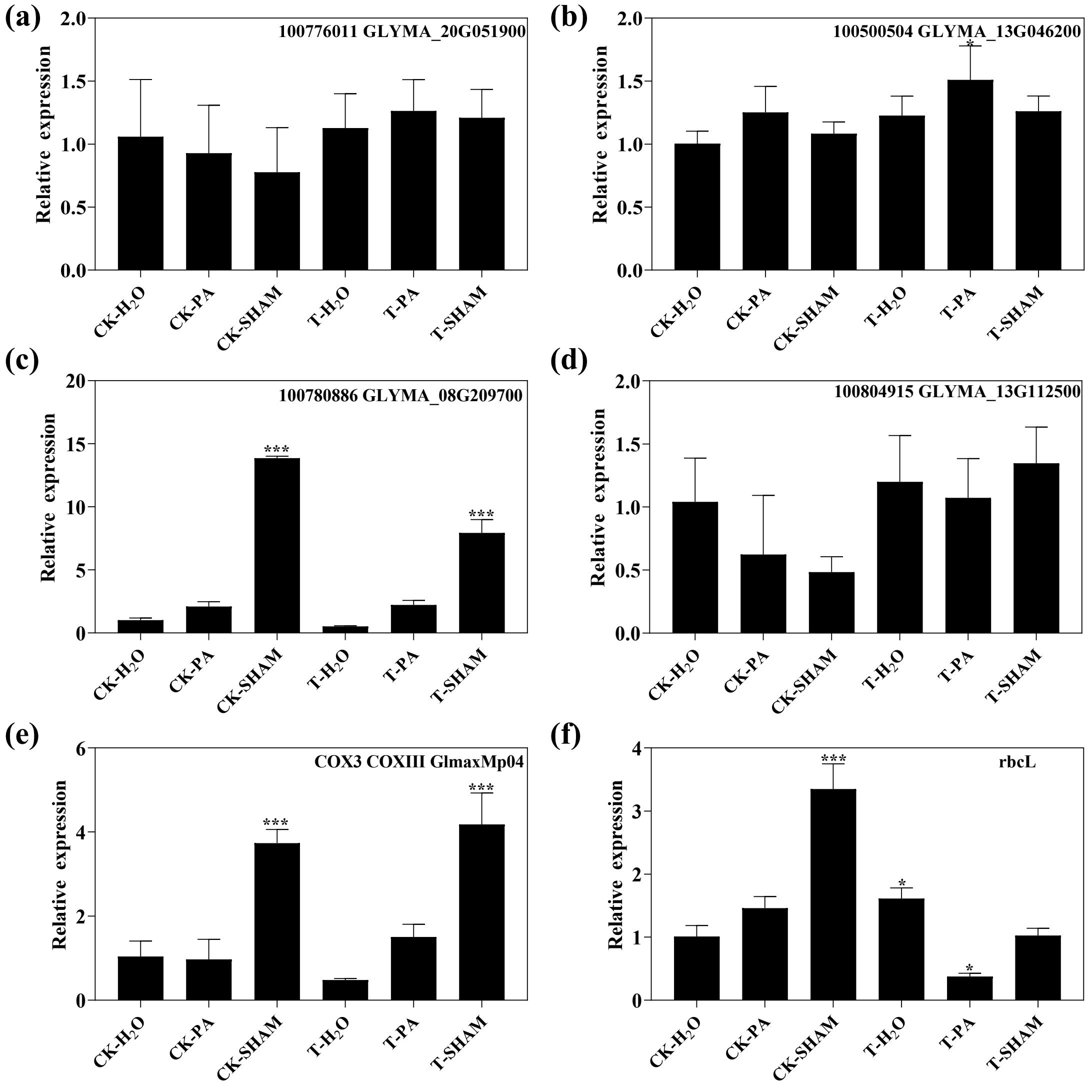

Supplementary Figure 6. The relative gene expression changes of DAPs were analysed by qRT-PCR for (a) 100776011 GLYMA_20G051900 (protein marker); (b) 100500504 GLYMA_13G046200; (c) 100780886 GLYMA_08G209700; (d) 100804915 GLYMA_13G112500; (e) COX3 COXIII GlmaxMp04 and (f) rbcL in *Robinia pseudoacacia* at high CO_2. Three biological replicates were analyzed, and the error bars represent SE. Asterisks indicate significant difference as determined by independent *t*-test. (* $P \leq 0.05$, ** $P \leq 0.01$, *** $P \leq 0.001$).
