# Supplementary material for: Physiological and Proteomic Responses of the Tetraploid Robinia pseudoacacia L. to High CO2 Levels
Source: Int J Mol Sci. 2024 May 11;25(10):5262. doi: 10.3390/ijms25105262 (PMC11121411; doi:10.3390/ijms25105262)
Supplement: Supplementary file 1 [file ijms-25-05262-s001.zip › Supplementary Tables.pdf]

Table S1. Sequences of the primers

Primer name	Primer sequence (5'-3')
18S-rRNA-F/R	TAGTTGGTGGAGCGATTTGTC<br>CAGAACATCTAAGGGCATCACAG
rbcL-F/R	TGAATGTCTTCGTGGGGGAC<br>AAACGGTCTCTCCAACGCAT
100776011-F/R	TGATGGGAATCAACGGCCAG<br>AGCACACTGTGAGATGGCAG
100500504-F/R	TACCGTGAGCACAACAGGTC<br>GCGATGAAGCTGATGCACTGK
100780886-F/R	TCGTGGGGCTTGTCTTTCTCTC<br>AAGGAAGCACCTGCTGTGTGT
100804915-F/R	GTGTGAAGACAGGTTTGCAGG<br>TTTGAACCATTGAACTCCACCTC
COX3-F/R	GCTACCGTTTCACTGGCTCT<br>ACGTGATGCTCCTTGGTCAG

Table S2. Candidate proteins responsive to high CO_2 treatment

Protein name	UniProtEntry
Ribulose bisphosphate carboxylase large chain (EC 4.1.1.39) (Fragment)	A0A0F6Y5S8/ C0J370/ I6QMJ2
Ribulose-1,5-bisphosphate carboxylase/oxygenase large subunit	A0A898CTM2
Photosystem II CP43 reaction center protein	A0A6H0EHP1
Photosystem II CP47 reaction center protein	A0A898CWR3/A0A1C7D4D5
Photosystem II CP44 reaction center protein	A0A898CTK1
Photosystem I P700 chlorophyll a apoprotein A2	A0A1C7D3U7/A0A1C7D4C6
Photosystem I P700 chlorophyll a apoprotein A1	A0A6H0EHR0/A0A1C7D3T6
Photosystem II protein D1	A0A898CTN3
Photosystem II D2 protein	A0A1C7D3T6/A0A1C7D3V4/ A0A898CVS6
Photosystem I subunit VII	A0A898CW14
Photosystem I reaction center subunit II	I3STB2
Cytochrome f	A0A1C7D3W5
Plastoquinol-plastocyanin reductase	I1LUB3/I3RZ73
Chlorophyll a-b binding protein	A0A0R0FRF8/I3SIW2
Photosystem I P700 chlorophyll a apoprotein A2	A0A898CW46
Photosystem I iron-sulfur center	A0A1C7D3Z6
Glutathione peroxidase	I1KP94/I3SK85
Glutathione-disulfide reductase	I3T078
Glutamine synthetase	A0A0R4J3P1
CPSase-sm-chain domain-containing protein	I1KBF7

Tau class glutathione S-transferase	K7KDM7
Allene oxide synthase	A0MAV5
Peroxidase (EC 1.11.1.7)	A0A0R0KJA5/ A0A0R0ERX9
Tau class glutathione S-transferase	C6T460/I1K5J3/C6TAT2
Glutamine synthetase (EC 6.3.1.2)	A0A0R0EVM7
Lactoylglutathione lyase (Glyoxalase I)	I1LR74
Uncharacterized protein	C6T2G1/ C6TAW8
Glutathione peroxidase	I1MX60/ C6SY48
Peroxidase (EC 1.11.1.7)	I1LHW6
Cytochrome c oxidase subunit 2 (EC 7.1.1.9)	P05491
Cytochrome b6	A0A898CTT0
Cytochrome b-c1 complex subunit Rieske, mitochondrial (EC 7.1.1.8)	C6THW5
3Cytochrome b-c1 complex subunit 7	I1MWP7
Cytochrome f	A0A0R0H975
NADH dehydrogenase subunit I	A0A898CVZ3
6-phosphogluconate dehydrogenase, decarboxylating (EC 1.1.1.44)	I1KWM7
NAD(P)H dehydrogenase (quinone) (EC 1.6.5.2)	A0A0R4J3V9/ A0A0R0GRT9/ I1LT63
NADH dehydrogenase subunit 7	A0A898CWS3
Chalcone synthase (EC 2.3.1.74)	I1LG10
Chalcone-flavonone isomerase family protein	C6SWQ7
Phenylalanine ammonia-lyase (EC 4.3.1.24)	I1NA96/A0A0R0KUR1
